# Sandwich Caregiving Among Nursing University Faculty Staff: A National Cross-Sectional Study of Prevalence and Organizational Support Needs

**DOI:** 10.7759/cureus.98339

**Published:** 2025-12-02

**Authors:** Yutaro Takahashi, Ryota Kumakura, Ruka Saito, Rie Okamoto, Koji Tanaka, Shizuko Omote

**Affiliations:** 1 Faculty of Health Sciences, Institute of Medical, Pharmaceutical and Health Sciences, Kanazawa University, Kanazawa, JPN

**Keywords:** caregivers, child care, cross-sectional studies, elder care, japan, nursing faculty, social support

## Abstract

Background: Although much research regarding the burdens faced by sandwich caregivers and effective coping strategies does exist, inter-study comparisons have been difficult, partly owing to inconsistencies in the definition of a sandwich caregiver. Therefore, this study aimed to investigate the prevalence and characteristics of sandwich caregiving among nursing university faculty staff, assess differences in support needs across sandwich caregiving, childcare-only, eldercare-only, and neither groups, and identify specific types of organizational and institutional support preferred by the sandwiched caregiving group.

Materials and methods: This cross-sectional descriptive study involved a secondary analysis of national survey data obtained in 2020 from 1,084 full-time nursing university faculty staff. Participants were categorized into four groups: sandwich caregiving, childcare-only, eldercare-only, and neither. Nineteen support needs items were assessed using a five-point Likert scale. Descriptive statistics and chi-square tests were applied to compare groups, with national estimates calculated.

Results: The prevalence of sandwich caregiving among nursing university faculty staff was 3.8%, corresponding to an estimated 349 individuals nationally. This prevalence was approximately 19 times higher than that in the general population and 2.7 times higher than that in married women. The sandwiched caregiving group demonstrated the highest demand rates across 11 items, with eight items exhibiting maximum differences of five percentage points or more between groups. Notably, demand for training on effective teaching methods during the COVID-19 pandemic reached 100% within this group. The sandwiched caregiving group expressed a greater need for organizational guidance and recommendations regarding research, education, and working practices.

Conclusion: Nursing university faculty staff experience substantially higher rates of sandwich caregiving than the general population and exhibit distinct support needs that prioritize organizational-level interventions over individual coping strategies.

## Introduction

Extensive research has generated knowledge regarding the multifaceted burdens faced by sandwich caregivers and the coping strategies they adopt. Sandwich caregivers, individuals who simultaneously provide care for both children and elderly parents, face unique challenges in balancing competing care responsibilities. A systematic review by Honda et al. [1] identified working hours, workplace flexibility, and partner support as factors influencing work-family conflict among sandwich caregivers, demonstrating that maintaining flexible work arrangements and ensuring social connections are important stress-coping strategies. However, challenges persist regarding research comparability. Hodgdon et al. [2] noted that inconsistencies in the definition of sandwich caregivers hinder inter-study comparisons, and Albertini et al. [3] reported that the prevalence of socially sandwiched caregivers, those actively providing support to both generations, varies considerably depending on the operational definition of support. Given these challenges, a more detailed understanding of sandwich caregivers' real-world experiences is necessary.

Japan presents a particularly salient context for examining this phenomenon, as it has one of the most rapidly aging populations in the world, with 28.8% of the population aged 65 years and older as of 2020 [4]. With increasing female workforce participation and trends toward later marriage and childbearing, the challenges faced by sandwiched caregivers have gained growing recognition [5].

According to the Cabinet Office and Government of Japan survey [5], approximately 0.2% of individuals aged 15 years and older engage in sandwiched caregiving, corresponding to roughly 250,000 people nationwide, with women providing care at nearly twice (1.96 times) the rate of men. Prior research focusing on married Japanese women indicates that those providing sandwiched care experience poorer subjective health than those without childcare or eldercare responsibilities [6], suggesting significant physical and mental burdens.

Qualitative research has documented Japanese women’s experiences of sandwiched caregiving, including feelings of “inability to fulfill the maternal role” and “having no one to talk to about caregiving pressures” [7], highlighting the disproportionate concentration of complex caregiving responsibilities among women.

However, empirical research on sandwich caregiving among nursing university faculty staff in Japan remains extremely limited. While international findings emphasize the importance of workplace cultural flexibility for supporting the well-being of sandwiched caregivers [1,3], evidence regarding the effectiveness of specific support measures in the Japanese nursing education context remains insufficient.

The global nursing profession predominantly comprises women who face particular difficulties in balancing caregiving responsibilities with professional duties. Studies show that nursing faculty staff experience significant work-family conflict, with factors such as working more than 40 hours per week significantly predicting high turnover intention [8]. Nursing faculty staff have described their experiences as an “unexpected journey,” reporting a lack of role clarity and insufficient preparation for academic roles within a work environment characterized by low salaries, excessive workloads, and limited institutional support [9].

In Japan, similar challenges exist. The nursing profession comprises approximately 92% women [10], and childcare and eldercare rank among the top reasons for nurse turnover [11,12]. Among nursing university faculty staff, the proportion of women is high at approximately 89% [13], and they face particular difficulties in balancing their home care responsibilities with their professional duties. While the nursing shortage is becoming increasingly severe, the number of unfilled nursing faculty positions is rising [13], with only 45.2% of faculty staff reporting that they "want to continue working at their current training institution" [14].

These challenges are compounded by cultural and gender norms. While gendered caregiving expectations contribute to women's disproportionate involvement in caregiving across many countries [15], in Japan, traditional norms that designate women as family caregivers remain particularly entrenched [6,7]. Despite the formal abolishment of the traditional family system, caregiving responsibilities are still concentrated among women, with deep-rooted gender role divisions persisting in Japanese society [7]. These expectations may create additional psychological burden for female nursing educators managing both professional and family care responsibilities, with critical implications for workforce retention in nursing education.

The COVID-19 pandemic further intensified these challenges for nursing educators globally. Faculty staff experienced significant guilt and dramatically increased workloads due to the rapid transition to online education [16,17]. For those managing both childcare and eldercare, the pandemic likely amplified their overlapping burdens-professional demands, student support responsibilities, and sandwiched caregiving for both children and aging parents. These extraordinary circumstances may have made the characteristics and challenges of sandwiched caregivers more pronounced, rendering pandemic-era data especially valuable for examining their work-life balance needs.

This study aimed to clarify the reality of sandwiched caregiving among nursing university faculty staff in Japan, examine whether there are differences in support needs of nursing faculty staff in sandwiched caregivers compared with others, and identify characteristics of support sought by nursing faculty staff in the sandwiched caregiving group.

## Materials and methods

Design

A cross-sectional descriptive study was conducted using secondary analysis of data from a national survey.

Setting and sampling

The data for this secondary analysis, "Survey of Members of the Japan Academy of Nursing Science (JANS): Impacts of COVID-19 on Research Activities and Support Expected From JANS, 2020 (First Survey)" was provided by the Social Science Japan Data Archive (SSJDA) at the Center for Social Research and Data Archives, Institute of Social Science, The University of Tokyo (https://doi.org/10.34500/SSJDA.1563) [18]. Permission for secondary data use was obtained from SSJDA following their standard data application procedures.

The original survey was distributed to 9,524 JANS members, and 1,532 responses were received (response rate: 16.1%) [18]. Participants were included in this analysis if they (1) answered "yes" to "Are you currently working full-time at a nursing university?" and (2) had complete responses to both childcare and eldercare status questions. Of the 1,532 respondents, 448 (29.3%) were excluded through sequential criteria, 278 (18.1%) did not meet the full-time nursing university employment criterion (245 answered "no" and 33 had missing responses), and 170 (11.1%) had missing data on childcare or eldercare status. This sample represents approximately 11.8% of the 9,227 nursing faculty at 290 Japanese universities [13]. Respondents with missing responses to demographic characteristics or the 19 support needs items were retained in the analysis, with missing values handled using listwise deletion for each specific analysis.

Exposure and comparator

The primary exposure of interest was sandwiched caregiving (a condition in which individuals responded "yes" to both "are you currently engaged in childcare/child-rearing?" and "are you currently providing care for elderly family members?"). The participants were classified into four groups: 1) sandwiched caregiving group, those who are responsible for both childcare and eldercare; 2) childcare-only group, those responsible for childcare only; 3) eldercare-only group, those responsible for eldercare only; and 4) neither group, those responsible for neither childcare nor eldercare. This binary classification approach is commonly used in Japanese double care research [5-7].

Data collection

Survey Instrument

As reported in a previous secondary analysis using this dataset [19], the instrument was developed by the JANS COVID-19 Nursing Research Countermeasures Committee based on established literature regarding COVID-19's impact on research communities and underwent pilot testing with JANS board members to establish face validity. While standard psychometric reliability indices were not formally reported, the appropriateness of this instrument as a secondary data source is supported by: (1) its systematic development process and expert validation, (2) the objective, factual nature of our core exposure variables (childcare and eldercare status), which are simple binary items requiring minimal psychometric validation, (3) archiving through the SSJDA, an established academic data repository at the University of Tokyo, and (4) alignment with validated binary classification approaches widely used in Japanese caregiving research [5-7].

Basic attributes

We collected available data on sex, age, position, degree, and research activity status (such as Grant-in-Aid for Scientific Research acquisition) from the original survey [18]. As a secondary analysis, our variable selection was limited to those collected in the original survey instrument.

Support needs

We assessed 19 items of support requests to the academic society under COVID-19 using a 5-point scale: “strongly want it to be implemented,” “want it to be implemented,” “somewhat want it to be implemented,” “neither,” and “no need to implement.”

Data analysis

Descriptive statistics were used to examine the distribution of basic demographic and professional attributes within each group. Differences in these attributes across groups were evaluated using χ² tests. For support needs comparison, the 5-point scale responses were reclassified by combining “strongly want it to be implemented,” “want it to be implemented,” and “somewhat want it to be implemented” into “want it to be implemented,” and “neither” and “no need to implement” into “no need to implement.” Descriptive analysis was conducted to identify patterns of support needs across the four groups. Demand rates were calculated for each item within caregiving groups, and groups were compared using χ² tests.

Missing data were handled using available case analysis, with sample sizes reported for each variable. No correction for multiple comparisons was applied, given the exploratory and descriptive nature of the study.

To facilitate national-level estimation, population data from the Japan Association of Nursing Programs at Universities were applied to calculate 95% CI for the prevalence of the sandwiched caregiving group. All statistical analyses were conducted using Stata 18.0 (StataCorp, College Station, TX, USA), with a significance level set at 0.05.

Ethical considerations

For the secondary analysis, we obtained de-identified individual-level data from SSJDA at the Center for Social Research and Data Archives, Institute for Social Science, University of Tokyo, for the "Survey of Members of the Japan Academy of Nursing Science (JANS): Impacts of COVID-19 on Research Activities and Support Expected From JANS, 2020 (First Survey)" [18]. As this study involved secondary analysis of de-identified archived data obtained under license from SSJDA and did not involve any direct contact with human participants, no additional ethical review was required. The study was conducted in accordance with established research ethics guidelines.

## Results

Characteristics of the sample

Of the 1,532 survey respondents, 1,084 met our inclusion criteria (full-time nursing university employment and complete caregiving status data). The breakdown was as follows: sandwiched caregiving group, 41 (3.8%); childcare-only group, 322 (29.7%); eldercare-only group, 132 (12.2%); and neither group, 589 (54.3%) (Table 1).

**Table 1:** Characteristics of nursing university faculty by four groups (n=1,084)* aKAKENHI (Grant-in-Aid for Scientific Research) is the major public competitive grant program for scientific research in Japan. *Total sample size; actual denominators vary by item due to missing data. PI, principal investigator.

		Sandwiched caregiving group (n=41)	Childcare-only group (n=322)	Eldercare-only group (n=132)	Neither group (n=589)	χ²	df	p-value
Sex	Male	2 (5.4)	24 (8.1)	5 (4.0)	56 (10.0)	5.894	6	0.435
	Female	35 (94.6)	267 (90.5)	117 (94.4)	498 (88.4)			
	Prefer not to answer	0 (0.0)	4 (1.4)	2 (1.6)	9 (1.6)			
Age	<35 years old	1 (2.7)	20 (6.7)	6 (4.8)	39 (6.9)	8.871	15	0.884
	36–45 years old	10 (27.0)	72 (24.0)	31 (24.8)	152 (26.9)			
	46–55 years old	17 (46.0)	100 (33.3)	46 (36.8)	197 (34.9)			
	56–65 years old	7 (18.9)	88 (29.3)	34 (27.2)	148 (26.2)			
	66 years old or older	2 (5.4)	14 (4.7)	4 (3.2)	17 (3.0)			
	Prefer not to answer	0 (0.0)	6 (2.0)	4 (3.2)	12 (2.1)			
Position	Professor	10 (27.0)	109 (36.7)	41 (33.1)	181 (32.1)	9.489	18	0.947
	Associate professor	10 (27.0)	62 (20.9)	31 (25.0)	119 (21.1)			
	Lecturer	10 (27.0)	56 (18.9)	2 (21.8)	114 (20.2)			
	Assistant professor	6 (16.2)	57 (19.2)	22 (17.7)	122 (21.6)			
	Teaching associate	1 (2.7)	8 (2.7)	1 (0.8)	13 (2.3)			
	Others	0 (0.0)	0 (0.0)	0 (0.0)	1 (0.2)			
	Prefer not to answer	0 (0.0)	5 (1.7)	2 (1.6)	14 (2.5)			
Academic degree	Doctor	18 (43.9)	154 (48.0)	71 (53.8)	294 (49.9)	12.534	12	0.404
	Master	23 (56.1)	158 (49.2)	57 (43.2)	273 (46.4)			
	Bachelor	0 (0.0)	4 (1.2)	1 (0.8)	18 (3.1)			
	Others	0 (0.0)	4 (1.2)	2 (1.5)	1 (0.3)			
	Prefer not to answer	0 (0.0)	1 (0.3)	1 (0.8)	2 (0.3)			
Acquisition of KAKENHI^a^ as a PI	Yes	20 (55.6)	160 (53.9)	64 (51.2)	278 (50.1)	1.352	3	0.717
	No	16 (44.4)	137 (46.1)	61 (48.8)	277 (49.9)			

Using these results, we estimated the national number of sandwiched caregiving nursing faculty staff using the total of 9,227 nursing faculty at Japanese universities [14], which yielded approximately 349 individuals (95% CI: 244-454), representing 3.8% (95% CI: 2.6%-4.9%) of all nursing faculty staff.

Regarding the basic attributes of the double-care group, 35 (94.6%) participants were women, exceeding the 88.4% in other groups. The age distribution showed that 17 (46.0%) participants were in the age group of 46-55 years, representing the largest proportion, followed by 10 (27.0%) participants in the 36-45 years age group. The academic positions were evenly distributed: 10 (27.0%) professors, 10 (27.0%) associate professors, 10 (27.0%) lecturers, and 6 (16.2%) assistant professors. Regarding the highest degree attained, 23 (56.1%) held a master’s degree and 18 (43.9%) held a doctoral degree. Regarding research activities, 20 (55.6%) had obtained a Grant-in-Aid for Scientific Research as principal investigators.

No statistically significant differences were observed among the four groups in terms of sex (χ²=5.894, df=6, p=0.435), age (χ²=8.871, df=15, p=0.884), position (χ²=9.489, df=18, p=0.947), academic degree (χ²=12.534, df=12, p=0.404), or grant-in-aid acquisition status (χ²=1.352, df=3, p=0.717).

Support needs comparison

We conducted a four-group comparison of support needs across 19 items. The results are presented in Table 2. Although no statistically significant differences were observed across any items (all p>0.05), important patterns emerged across several items.

**Table 2:** Four-group comparison of support needs among nursing university faculty (n=1,084)* *Total sample size; actual denominators vary by item due to missing data. ICT, information and communications technology; JANS, Japan Academy of Nursing Society.

	Sandwiched caregiving group (n=41)	Childcare-only group (n=322)	Eldercare-only group (n=132)	Neither group (n=589)	χ²	df	p-value
1. Research grants related to the COVID-19 pandemic	26/41 (63.4)	231/319 (72.4)	86/130 (66.2)	406/584 (69.5)	2.664	3	0.446
2. Financial assistance for individuals unable to start or continue studies abroad due to the COVID-19 pandemic	26/41 (63.4)	193/316 (61.1)	72/131 (55.0)	344/582 (59.1)	1.731	3	0.63
3. Collaboration on surveys by JANS members (requests and distribution of survey forms)	28/40 (70.0)	220/321 (68.5)	89/128 (69.5)	389/577 (67.4)	0.339	3	0.953
4. Making JANS-conducted surveys available as open-source data	32/41 (78.0)	269/321 (83.8)	108/131 (82.4)	483/580 (83.3)	0.911	3	0.823
5. Expanding online seminar and workshop opportunities	40/41 (97.6)	308/319 (96.6)	125/129 (96.9)	549/579 (94.8)	2.447	3	0.485
6. Increasing online opportunities for interaction and consultation among JANS members (forums, mailing lists, and private social media groups)	29/41 (70.7)	222/318 (69.8)	87/131 (66.4)	402/576 (69.8)	0.652	3	0.884
7. Establishing online journal clubs	26/39 (66.7)	224/316 (70.9)	83/130 (63.8)	402/580 (69.3)	2.266	3	0.519
8. Organizing online research meetings	31/40 (77.5)	225/312 (72.1)	84/131 (64.1)	426/585 (72.8)	4.761	3	0.19
9. Building online systems for individual research consultation	28/41 (68.3)	216/320 (67.5)	73/131 (55.7)	366/580 (63.1)	6.066	3	0.108
10. Sharing case studies of joint research conducted remotely and effectively	29/41 (70.7)	256/313 (81.8)	94/129 (72.9)	468/579 (80.8)	7.072	3	0.07
11. Sharing examples of research successfully conducted while working from home during the COVID-19 pandemic	24/38 (63.2)	220/321 (68.5)	83/130 (63.8)	407/578 (70.4)	2.762	3	0.43
12. Sharing case studies of management that effectively addressed the impact of the COVID-19 pandemic	31/39 (79.5)	249/319 (78.1)	95/128 (74.2)	446/574 (77.7)	0.946	3	0.814
13. Providing training on research methods that can be implemented during crises, including the COVID-19 pandemic	35/41 (85.4)	276/315 (87.6)	109/131 (83.2)	483/576 (83.9)	2.613	3	0.455
14. Providing training on effective teaching methods used during the COVID-19 pandemic	41/41 (100.0)	303/319 (95.0)	118/128 (92.2)	537/576 (93.2)	4.42	3	0.22
15. Building networks to promote continuity between research, teaching, practice, and policy during major health crises, including the COVID-19 pandemic	35/41 (85.4)	270/317 (85.2)	111/131 (84.7)	493/578 (85.3)	0.028	3	0.999
16. Offering recommendations on research during the COVID-19 pandemic to organizations that members are affiliated with	33/40 (82.5)	242/319 (75.9)	97/130 (74.6)	444/577 (76.9)	1.195	3	0.754
17. Offering recommendations on education during the COVID-19 pandemic to organizations that members are affiliated with	35/41 (85.4)	262/317 (82.6)	104/129 (80.6)	460/577 (79.7)	1.683	3	0.641
18. Offering recommendations on working styles during the COVID-19 pandemic to organizations that members are affiliated with	32/39 (82.1)	245/315 (77.8)	98/130 (75.4)	449/572 (78.5)	0.983	3	0.805
19. Providing recommendations to promote ICT proficiency among educators to organizations that members are affiliated with (e.g., employment of ICT support staff)	32/40 (80.0)	259/317 (81.7)	106/131 (80.9)	464/577 (80.4)	0.238	3	0.971

The double-care group demonstrated the highest demand rates among the four groups for all 11 items. Of these, eight items exhibited maximum differences of 5 percentage points or more between groups: financial assistance for individuals unable to start or continue studies abroad due to the COVID-19 pandemic (63.4% vs. 55.0%-61.1%, χ²=1.731, df=3, p=0.630), forming online research meetings (77.5% vs. 64.1%-72.8%, χ²=4.761, df=3, p=0.190), establishment of online systems for individual consultation related to research (68.3% vs. 55.7%-67.5%, χ²=6.066, df=3, p=0.108), sharing cases of study management that effectively addressed the impacts of the COVID-19 pandemic (79.5% vs. 74.2%-78.1%, χ²=0.946, df=3, p=0.814), training on effective teaching methods during the COVID-19 pandemic (100.0% vs. 92.2%-95.0%, χ²=4.420, df=3, p=0.220), recommendations on research practices during the COVID-19 pandemic for affiliated organizations (82.5% vs. 74.6%-76.9%, χ²=1.195, df=3, p=0.754), recommendations on educational practices during the COVID-19 pandemic for affiliated organizations (85.4% vs. 79.7%-82.6%, χ²=1.683, df=3, p=0.641), and recommendations on flexible working styles during the COVID-19 pandemic for organizations that members are affiliated with (82.1% vs. 75.4%-78.5%, χ²=0.983, df=3, p=0.805).

Regarding training on effective teaching methods during the COVID-19 pandemic, only the double-care group exhibited a 100% demand rate, exceeding that of the other groups (92.2%-95.0%) by 7.8 percentage points.

## Discussion

Albertini et al. [3] reported a 2%-8% prevalence of social sandwiched caregiving status across 17 European countries. The 3.8% rate observed among nursing university faculty staff in this study falls within this international range. By contrast, when compared to the general Japanese population (0.2%) and married women aged 20-59 years (1.4%) [5,6], nursing educators experienced approximately 19 and 2.7 times higher rates, respectively. This disparity highlights that nursing educators face substantially elevated risk of sandwich caregiving compared to the broader Japanese population, likely reflecting the intersection of professional demands, delayed childbearing, and the predominantly female composition of the nursing workforce.

Research has documented that nursing faculty staff experience significant work-family conflict, with heavy workloads significantly predicting high turnover intention [8]. Faculty also report a lack of role clarity, insufficient preparation, and work environments characterized by excessive workloads and limited institutional support [9]. In Japan, approximately 89% of nursing university faculty staff are women [13], bearing multiple professional responsibilities, including education, research, clinical instruction, and university administration. These occupational characteristics make nursing educators structurally prone to sandwiched caregiving situations.

Research underscores the importance of organizational support in promoting work-life balance. Honda et al. [1] demonstrated that sandwiched caregivers experience “time-based conflict,” necessitating improvements in “schedule control,” while Boyczuk and Fletcher [20] revealed that caregiving burdens fluctuate dynamically depending on parental health and children’s circumstances.

Nursing faculty in the sandwiched caregiving group in this study demonstrated a clear preference for organizational-level support structures. The 100% request rate for “training on effective teaching methods during the COVID-19 pandemic” and the consistently high demand for recommendations to affiliated organizations underscore an urgent need for institutional interventions, marking a departure from conventional reliance on individual coping strategies [21,22].

The pandemic further amplified these support needs, particularly among faculty staff in the sandwiched caregiving group. It exacerbated pre-existing structural challenges, as nursing educators navigated increased workloads and caregiving demands simultaneously. Faculty staff reported significant guilt and dramatically elevated workloads due to the rapid shift to online education [16,17], with some describing the experience as requiring “double or triple the number of hours to teach and support students compared to previous semesters” [17], all while managing intensified family care responsibilities.

These demands reflect complex, multifactorial risks specific to nursing educators in the sandwiched caregiving group. Research shows that individuals managing both childcare and eldercare exhibit poorer subjective health outcomes [6]. Among nursing educators, the psychological burden is intensified by a disconnect between professional caregiving knowledge and personal caregiving realities. Moreover, cultural and gender norms that designate women as primary caregivers [15] further amplify this burden for female faculty staff.

In Japan, feelings of guilt and isolation, such as “inability to fulfill the maternal role” and “having no one to talk to about caregiving pressure” [7], are particularly pronounced among nursing educators who teach care theory. This is likely due to heightened cognitive dissonance between their professional ideals, societal expectations, and lived caregiving experiences. When eldercare responsibilities are layered onto an existing “sense of inadequacy in both work and childcare” [23], the psychological toll becomes even more significant. Adverse outcomes associated with workaholism, such as decreased job performance, burnout, family conflict, sleep disturbances, and chronic stress, are more likely to manifest as time and emotional reserves are depleted [24].

Faculty staff requests for “online research meetings” and institutional support can be interpreted as rational strategies to manage time constraints and fluctuating caregiving demands. Liu et al. [25] demonstrated that caregivers whose needs were met experienced significantly reduced depressive symptoms, reinforcing the value of targeted organizational interventions. However, institutional support alone may be insufficient. The caregiving burden is also shaped by broader societal systems, including public childcare and eldercare infrastructure, and national work-life balance policies. Addressing these challenges requires multilevel interventions that integrate organizational reforms within educational institutions and structural improvements in social support systems.

Research consistently reports high turnover intentions among nursing faculty staff, with heavy workloads serving as a major predictor [8]. The global shortage of nursing faculty staff remains a pressing concern. In the United States, the American Association of Colleges of Nursing reported a national faculty vacancy rate of 7.8% in 2023, which prevented more than 65,000 qualified applicants from entering nursing programs [26]. In Japan, the number of unfilled nursing faculty positions continues to rise [13], and only 45.2% of nursing educators wish to remain at their current institutions [14]. The estimated 349 faculty members in the sandwiched caregiving group nationwide pose serious policy concerns regarding educational quality and human resource retention.

These workforce challenges extend to physician-researchers, revealing systemic issues across healthcare professions. Early-career physician-scientists experience substantial burnout, with female gender associated with higher personal burnout, though workplace resources and mentorship serve as protective factors [27]. Female physicians in basic sciences report greater satisfaction when organizational flexibility accommodates caregiving [28], and female medical students prioritize work-life balance in career choice [29]. The emphasis on workplace resources and flexibility among physician-researchers mirrors the strong orientation toward organizational support demonstrated by nursing educators in this study, underscoring that without adequate support, female professionals across healthcare disciplines face substantial burden from overlapping research, education, and caregiving responsibilities.

The full participation (100%) in pandemic-related teaching training demonstrates remarkable professional commitment despite substantial caregiving demands. However, without adequate institutional support, time pressures may compromise the quality of nursing education. Evidence shows that nursing staff turnover is strongly shaped by life stages: “marriage,” “pregnancy and childbirth,” and “childcare” in the 30s-40s, and “family health and eldercare” in the 50s [11]. The identified “sandwiched caregiving” period (mainly 36-55 years old, with 46.0% aged 46-55 years) coincides with the peak turnover risk among core personnel. If 349 faculty members were to leave, the resulting replacement costs and decreased student enrollment could further intensify the national nursing shortage.

This global pattern highlights that the challenges identified are systemic within nursing education. The support needs revealed emphasize the need to shift from individual-level approaches to integrated organizational strategies. Recommendations for research, education, workstyle reform, and online research support are crucial for sustaining nursing education quality and safeguarding essential human resources.

Taken together, these findings reveal that sandwich caregiving among nursing faculty is not merely an individual challenge but a structural issue embedded within the intersection of demographic trends, professional characteristics, and cultural norms. The substantially higher prevalence among nursing educators compared to the general population, combined with the observed preference for organizational-level support, suggests that effective interventions must address institutional policies and workplace culture rather than focusing solely on individual coping mechanisms. Addressing this structural challenge requires systemic organizational responses; without such fundamental changes, nursing education institutions risk losing the experienced educators they can least afford to lose.

Study limitations and the future

This study has several limitations. First, selection bias may limit representativeness. The original survey achieved a 16.1% response rate (1,532 of 9,524 JANS members), and our final analytical sample of 1,084 full-time faculty represents approximately 11.8% of Japan's nursing faculty population [13]. The exclusion of 170 respondents (11.1%) with missing caregiving status data may have disproportionately excluded individuals managing complex care situations. Additionally, non-JANS members, non-respondents, and faculty experiencing severe caregiving burdens that prevented participation may be underrepresented, potentially leading to underestimation of the true prevalence and intensity of support needs. JANS membership is voluntary, and non-members may differ systematically in research engagement, institutional support, or caregiving responsibilities. These selection factors suggest that our findings likely represent a conservative estimate of the challenges faced by nursing faculty in sandwiched caregiving situations. However, the sample size was sufficient for examining prevalence patterns and group differences.

Second, this cross-sectional secondary analysis has methodological limitations. As a descriptive study, our analysis did not adjust for potential confounders such as age, academic rank, institutional type, or household structure, which may influence both caregiving arrangements and support preferences. Our binary classification captured the presence of dual caregiving responsibilities but did not assess caregiving intensity, duration, or care recipient characteristics. While this approach aligns with established methodologies in the field [4-6], individuals with varying levels of care burden may have different support needs. Additionally, the study was conducted during the COVID-19 pandemic and may reflect pandemic-specific conditions. Nevertheless, the pandemic exposed the vulnerabilities of nursing faculty staff in the sandwiched caregiving group, providing valuable insights applicable to both crisis and routine circumstances. The core challenges identified, time constraints, organizational support needs, and psychological burden, persist beyond pandemic settings but tend to intensify during crises. Longitudinal studies incorporating measures of caregiving intensity are needed to better understand how care burden moderates workplace support requirements, and to develop and validate institutional support frameworks that assess the effects on health, job satisfaction, and retention intentions among nursing faculty staff in the sandwiched caregiving group.

## Conclusions

This study identified a substantial prevalence of sandwiched caregiving among Japanese nursing university faculty staff (3.8%, with a national estimate of 349 individuals), highlighting their distinctive support needs. Faculty members in the sandwiched caregiving group exhibited a strong preference for organizational support focused on “recommendations to institutions regarding research, education, and workstyle reform,” in contrast to conventional individual-level measures. These findings reflect multifaceted risks, including internal conflict arising from discrepancies between professional knowledge and lived reality, time pressures, fluctuating caregiving demands, and culturally embedded gender norms that heighten psychological strain among female nursing educators.

Similar challenges are evident internationally. In the United States, a 7.8% nursing faculty vacancy rate has prevented over 65,000 qualified applicants from entering nursing programs. To sustain educational quality and prevent human resource loss, a shift from individual-focused interventions to integrated organizational strategies is essential. Institutional support measures, such as flexible work systems, protected research time, and reduced teaching loads, are vital to maintaining nursing education’s social mission and addressing the global shortage of nursing faculty staff.
